# One health–sustainability intersections: an umbrella systematic review with a new integrated definition of sustainability and a meta-conceptual framework

**DOI:** 10.1186/s42522-025-00187-z

**Published:** 2025-12-27

**Authors:** Osman Ahmed Dar, Melika Akhbari, Ali Akhbari, Hassaan Zahid, Max Claron, Neil Spicer, Hadjer Nacer, Mishal Khan

**Affiliations:** 1 Global Public Health Directorate, United Kingdom Health Security Agency, London, UK; 2 The GKT School of Medical Education, King’s College London, London, UK; 3 Imperial College Medical School, Imperial College, London, UK; 4 Medicins Sans Frontiers, Juba, South Sudan; 5 Health and Environment Programme Manager, Friendship France, Paris, France; 6 London School of Hygiene and Tropical Medicine, London, UK; 7 Faculty of Public Health, London, UK

**Keywords:** Sustainability, One health, Global health security, Analytical framework, Conceptual framework, Integrated framework systematic review, Integrated definition, Human-animal-environment interface

## Abstract

This umbrella systematic review synthesizes existing conceptual approaches to sustainability across human, animal, and environmental health disciplines, framed within the One Health paradigm. The study aims to develop a conceptual framework for analysing sustainability across One Health initiatives, interventions, and programs. The review included 38 studies that met the inclusion criteria, focusing on definitions, conceptual frameworks, and analytical frameworks of sustainability. Deductive thematic synthesis revealed key determinants and characteristics of sustainability, highlighting both similarities and differences across sectors. Across the 38 included reviews, we identified characteristics of sustainability existing along a process-outcomes continuum which we further grouped into fifteen process-focused characteristics, and outputs and outcomes that were categorized across a variety of contexts including acute emergency or humanitarian settings as well as within a stable macroeconomic and social construct. The review identified that sustainability has over time become more well defined and evaluated within the human health sector, with fewer review studies addressing it for animal and environmental health. Multisectoral studies often integrate the Brundtland Commission’s definition of sustainability, emphasizing social, economic, and environmental dimensions. The findings also highlight the importance of equity, resilience, and systems thinking in sustainability assessments. The analysis in our review underscores the need for a more holistic and integrated approach to sustainability that considers the interconnectedness of human, animals and ecosystems and that addresses the co-benefits and trade-offs across sectors. In our synthesis these concepts were coded from included reviews and mapped onto both the integrated definition and the meta-conceptual framework, ensuring they were structurally embedded into the proposed approach. A new integrated definition of sustainability is proposed, emphasizing quality-assured systems, services and interventions that are just and equitable, while being socially, economically, and environmentally viable, adaptable over time, and optimised for health benefits across humans, animals, and ecosystems without compromising future generations’ well-being. The accompanying evidence derived meta-conceptual framework serves as a normative guide for analysing sustainability in One Health contexts, adaptable across varying scales and health challenges. It is intended for use by policymakers, implementers and evaluators working on multisectoral programmes in human, animal and environmental health, particularly where a trade-off or co-benefit analysis is needed and where integrated approaches are being developed or scaled. Our study offers a robust evidence-based foundation for designing, assessing and implementing sustainable practices across One Health initiatives. Future research should focus on validating and further refining this framework through empirical studies across sectors and diverse One Health contexts. Particular attention should be given to addressing the gaps in sustainability assessments within the animal and environmental health sectors. As global health challenges continue to evolve, fostering a shared understanding of sustainability and promoting cross-sectoral collaboration will be essential for developing resilient and effective health interventions. In an era marked by increasing environmental pressures and shifting geopolitical landscapes, the principles of sustainability and One Health are more relevant than ever. This review contributes to the ongoing discourse on sustainability, providing a roadmap for stakeholders to navigate the complexities of multisectoral collaboration and ensure lasting impacts on global health and well-being across ecosystems.

**Supplementary information:**

The online version contains supplementary material available at 10.1186/s42522-025-00187-z.

## Introduction

Multisectoral perspectives on health across species—initially advanced under banners such as One Health and EcoHealth - have moved from conceptual discussion to mainstream policy during the past three decades. Early comparative work underscored that these approaches share a core premise: population health is inseparable from the integrity of ecosystems and the well-being of animals that share those ecosystems with humans [1]. Building on this premise, the Quadripartite One Health Joint Plan of Action 2022–2026 now provides countries with a detailed operational template that combines governance principles, priority technical areas, and monitoring benchmarks [2]. Parallel scholarship on Planetary Health further situates human flourishing within planetary biophysical limits, emphasising that environmental degradation undermines long-term health gains [3]. Analysis also highlights substantial overlap among these paradigms, suggesting an opportunity for unified research and implementation frameworks [4].

Adoption, however, of these multisectoral approaches to health has been uneven. For example, systematic evidence syntheses indicate that capacity for truly integrated surveillance and response remains limited, particularly in low- and middle-income settings [5]. Regional governance analyses similarly highlight fragmented mandates and financing streams across human, animal, and environmental health agencies [6]. These structural deficiencies have become salient in ongoing negotiations for a WHO-brokered pandemic agreement, where multisectoral coordination and equitable benefit-sharing are both prominent but contentious clauses [7]. Financing platforms—most notably the World Bank’s Pandemic Fund—offer new resources, yet their disbursement criteria still rely on measurable deliverables that countries struggle to define across sectors [8]. Critically however, both the Pandemic Agreement and the Pandemic Fund adopt and endorse the One Health approach.

To better support One Health programme design and implementation, recent analysis has called for metrics that extend beyond pathogen-specific outcomes to include social justice, ecological resilience, and economic viability [9]. The widely adopted and recognised definition of One Health broadly frames the approach as “sustainable and equitable health for humans, animals, and ecosystems achieved through transdisciplinary collaboration” [10]. However, this framing underscores a key analytic gap: ‘sustainability’ itself remains poorly defined and conceptualised across the One Health literature.

Sustainability science, rooted in socio-ecological theory, views health systems as complex adaptive entities shaped by feedback across temporal and spatial scales [11]. Yet sustainability discourse in global health has long been guided by the Brundtland Commission’s inter-generational equity principle [12] and, more recently, by the Sustainable Development Goals, which formalise environmental, economic, and social dimensions of progress [13]. Much of the health-related review literature has focused on this three-pillar model and while it dominates sustainability discourse and forms the focus of this paper, it is important to acknowledge alternative interpretations of the concept exist outside of the health sector. Also, despite these guiding texts, systematic reviews of programme evaluations reveal substantial heterogeneity in how sustainability is defined, measured, and reported. In parallel, the health resilience literature has expanded rapidly, often using similar language around adaptability, continuity and system functioning. However, resilience is typically oriented towards the capacity to absorb and recover from shocks, whereas sustainability is more explicitly concerned with long-term viability and inter-generational equity. The partial overlap and inconsistent use of these concepts across sectors further complicates efforts to develop shared analytical frameworks. Early constructs of the concept of sustainability emphasised continuation of funding and activities [14], while subsequent analyses incorporated health-system integration and institutional ownership [15]. In the nutrition and food-system literature, sustainability assessments increasingly link dietary patterns to ecological footprints and public-health outcomes [16]. Meanwhile, some implementation-science scholars have adopted a multi-attribute concept that incorporates continued benefits, organisational capacity, and adaptation to context [17].

Outside the human-health literature, reviews of livestock and agricultural systems show that sustainability envelopes economic viability, animal welfare, and ecological stewardship, yet methodological approaches remain disparate [18]. For example, in some veterinary and livestock systems literature, sustainability is framed primarily in terms of maintaining productive herds and disease control over time, whereas in environmental and ecosystem health studies it may emphasise restricting or reshaping human and animal use of landscapes to protect biodiversity and ecological integrity. These differences can lead to tensions in how trade-offs are described and which outcomes are prioritised across sectors. [16, 18] Recent appraisals of global health security indices observe that existing tools under-represent environmental and veterinary domains, thereby limiting their usefulness for integrated capacity assessments [19]. Consolidated evidence on attributes that enable programmes, services, or policies to remain effective over time—and across sectors—remains scarce. Where such attributes are described, they are rarely operationalised into conceptual or analytic frameworks suitable for monitoring and evaluation of sustainability [20].

Against this backdrop, the present umbrella systematic review pursues three objectives:

Conceptual consolidation—to map and compare definitions and theoretical constructs of sustainability used across human, animal, and environmental health scholarship;

Determinant synthesis—to catalogue attributes or characteristics of sustainability repeatedly linked to the continuation, maintenance or adaptation of programmes, interventions, services or systems; and

Framework appraisal—to identify and discuss tools, indicators, and analytical frameworks currently deployed to assess sustainability in multisectoral contexts.

By integrating evidence across disciplines, the review seeks to provide an empirically grounded definition and conceptual framework for sustainability that can inform the design, monitoring, and evaluation of One Health initiatives and, more broadly, guide investments aimed at durable health gains across sectors.

## Methods

### Design rationale and review approach

This umbrella review adopted a systematic methodology to explore how sustainability has been defined and conceptualised across human, animal, and environmental health sectors, with a particular focus on its multisectoral application within One Health paradigms. A preliminary exploration of the literature highlighted that most existing reviews are disciplinary in scope—primarily focusing on human health—and rarely engage with sustainability from a truly integrative, cross-sectoral standpoint. To address this gap, we employed an overarching review of review studies, following a structured and protocol-driven process. An umbrella review was selected, rather than a meta-narrative or realist synthesis, because our aim was to integrate and compare conceptual and analytical approaches across heterogeneous review types and disciplines, rather than reconstruct narrative traditions within a single field or develop context–mechanism–outcome configurations for specific interventions. The umbrella design allowed us to treat existing reviews as the unit of analysis and to map points of convergence and divergence in how sustainability is defined, operationalised and evaluated across human, animal and environmental health. Comprehensive details regarding the rationale, data management, and analytic procedures are available in the published protocol which is available open-access [21].

### Literature identification and selection process

A structured literature search was implemented across four electronic databases—Medline, Embase, Global Health, and Web of Science Core Collection—encompassing all records up to 14 April 2024. Search terminology was informed by prior systematic reviews [14–16] and expanded using strategies developed by Lennox et al. [22] and Zurynski et al. [23]. A tabulated breakdown of search terms and database-specific strategies is provided in the open-access protocol document [21]. For ease of reference the specific search strategy used is described in Box 1 below.

**Box 1:** Search strategy

SOURCE	SEARCH TERMS			SEARCH STRATEGY
	A	B	C	
Medline (Ovid); Embase (Ovid); Global Health (Ovid); Web of Science Core Collection (Web of Science, Clarivate Analytics);	systematic review; narrative review; scoping review; meta-analysis; literature review; critical review; mixed methods review;	One Health; EcoHealth; Planetary Health; One Medicine; Animal health; Human health; Public health; Environmental public health; Ecological health; Ecosystem health; Environmental health; agricult$ health; wildlife health; aquacult$ health; healthcare delivery; health program$; health service; healthcare system; health intervention; health system; healthcare; health promotion;	resilience; viability; institutionali$; routini$; durability; stability; Long-term implementation; sustain$; adaptive management	All terms listed within “A” separated with OR; all terms listed in “B” separated with OR; all terms listed in “C” separated with OR and then A + B + C terms combined with AND; Words should be in title/abstract

To qualify for inclusion, studies had to be review-based (systematic, scoping, narrative, or integrative) and address either conceptual definitions or analytical constructs of sustainability in human, animal, or ecosystem health contexts. Reviews that examined sustainability as a primary analytical theme, either through theoretical frameworks or evaluative metrics, were prioritised. Studies were excluded if they referenced sustainability only marginally, lacked health-related relevance, were opinion-based without formal synthesis methods, or were not accessible in full-text English (see protocol paper for full details of inclusion and exclusion criteria).

A supplementary set of reviews that evaluated sustainability as a primary outcome of health-related programmes or interventions—particularly in human and environmental health—were identified and examined separately in a limited secondary analysis. These studies were used to understand the applied use of sustainability frameworks but were not included in the main synthesis or subjected to quality appraisal.

### Screening, appraisal, and data extraction

The Preferred Reporting Items for Systematic Reviews and Meta-Analyses (PRISMA) guidelines informed our documentation and flow of included records. Title and abstract screening was undertaken independently by four reviewers (OD, MA, AA, MC), followed by iterative consensus meetings to resolve any interpretive discrepancies. Full-text reviews were completed by two reviewers (OD, MA), who initially aligned on coding categories and data elements prior to extraction. Disagreements were resolved through discussion to ensure interpretive coherence. The design of the data abstraction tools and screening logic are fully delineated in the protocol [21].

All review articles dedicated to the conceptual interrogation of sustainability were assessed for methodological quality using the AMSTAR 2 instrument [24]. We did not exclude studies solely on the basis of lower AMSTAR 2 ratings, nor did we apply formal quantitative weighting by quality, because the conceptual and heterogeneous nature of the review literature meant that potentially important dimensions of sustainability were sometimes articulated in methodologically weaker but substantively rich papers. Instead, AMSTAR 2 assessments were used descriptively to characterise the overall rigour of the evidence base across sectors and to inform a cautious interpretation of themes that were supported predominantly by lower-quality reviews.

### Analytical strategy and thematic synthesis

Due to the inherently diverse and conceptual nature of the included studies, we employed a qualitative, thematic synthesis approach grounded in established procedures for synthesising non-quantitative data [25–27]. Text excerpts describing determinants, characteristics, or conceptual underpinnings of sustainability were extracted and charted into a thematic matrix. Each excerpt was additionally tagged according to the sectoral focus of the source review (human health, animal health, environmental/ecosystem health, or multisectoral), which enabled us to compare how similar themes were framed across sectors without imposing differential weights on particular domains. Initial coding categories were based on the review’s analytic aims, with further codes generated deductively in response to emerging patterns in the data.

An iterative, interpretive analysis was used to identify sectoral convergences and divergences in how sustainability is defined, operationalised, and evaluated. Themes were grouped into process-oriented and outcome-oriented domains, and similarities and sector-specific variations were systematically compared across human, animal, and environmental health literature. Particular attention was paid to intersections, trade-offs, and framing distinctions between the sectors.

Findings from this synthesis were used to construct a definition and meta-conceptual framework for sustainability applicable across One Health contexts. Full details of methods relating to analysis and synthesis are available in the published protocol [21].

## Results

### Study selection

**Fig. 1:**
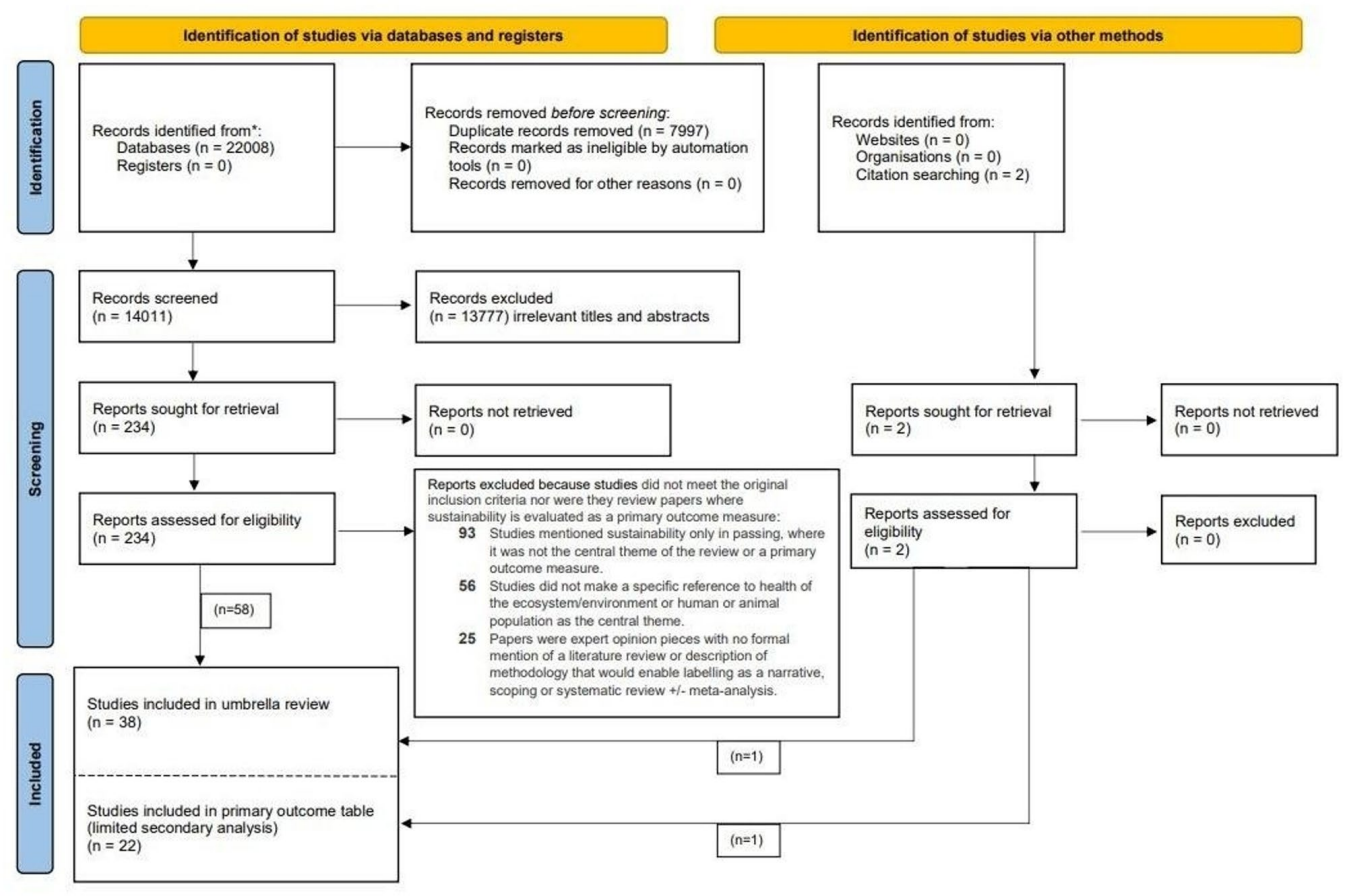
PRISMA flow chart – search results

A total of 14,011 studies were screened after duplicates were removed. At the title/abstract review stage, 13,777 out of the 14,011 were excluded, leaving 234 studies progressing to full-text review. An additional two suitable studies for full-text review were identified through citation searching (see Fig. 1). Of the 236 potentially eligible studies, 38 studies were included in our review for full data extraction, quality appraisal, analysis and synthesis where the main focus of the studies were conceptual or analytical approaches to sustainability and its characteristics and determinants. A summary of the findings for each study and the identified determinants and indicators for assessing sustainability are provided in appendix 1 [14–18, 20, 22, 23, 28–57]. The main reasons for exclusion at full-text review were that the study did not assess sustainability or characterize determinants of sustainability as the main focus of the review and/or that they were not explicitly concerned with the health of humans and/or animals and/or ecosystems/environment as the central theme of enquiry but rather focused on other issues and sectors unrelated to health directly (e.g. education, digital technologies, construction industry etc). No additional sector-specific attrition was recorded because exclusions were not made on the basis of human, animal or human health sectors.

Separately, a further 22 review studies [58–79] were also identified from the list of papers undergoing full-text review, where evaluations of sustainability for particular health-related interventions/services or strategies were the main primary outcome of interest. These have been summarized in appendix 2 and include a tabulated description of determinants, analytical frameworks and indicators used in each study for evaluating sustainability as a primary outcome. While these studies were not included in the final list of included studies for this umbrella systematic review as they were not conceptual in nature, they provide an insightful summary of what sustainability frameworks are most commonly being used for in evaluation reviews, and for what types of health issues and sectors these reviews have been conducted in the published literature.

### Review studies characteristics and quality appraisal

**Fig. 2:**
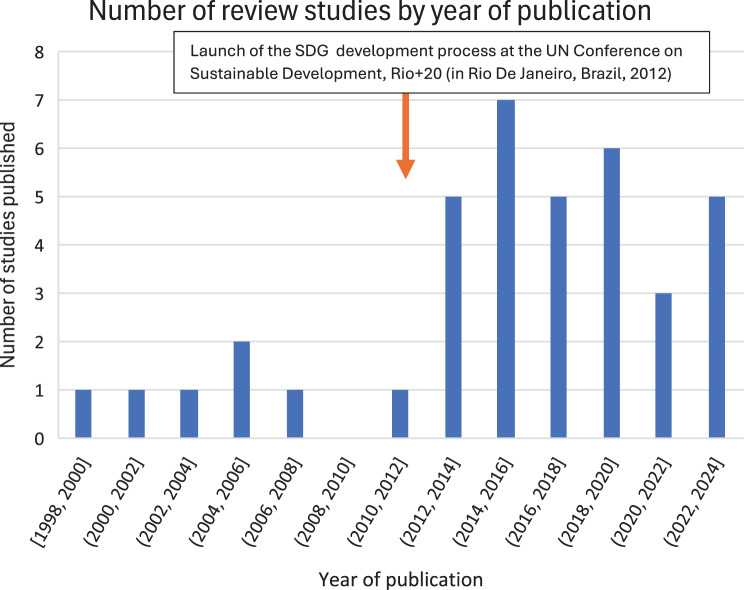
Review studies conceptualizing sustainability across human and/or animal and/or environmental health identified by year of publication

Shediac-Rizkallah and Bone’s seminal review study on sustainability published in 1998 is the first review study on sustainability and health identified through our systematic search. (55) As illustrated by Figure 2 below, there were relatively few reviews published over the next 15 years, with almost all reviews exclusively focused on sustainability within the human health sector alone. From 2013 onwards there was a growing academic and research interest in the subject, with more review studies on sustainability published every year, as well as a wider range of topics of focus, extending beyond human health to address the animal health and ecosystems/environment health sectors as well. This may well have coincided with the launch of the development process for the SDGs at the landmark UN Conference on Sustainable Development in Rio De Janeiro, Brazil in 2012.

**Fig. 3:**
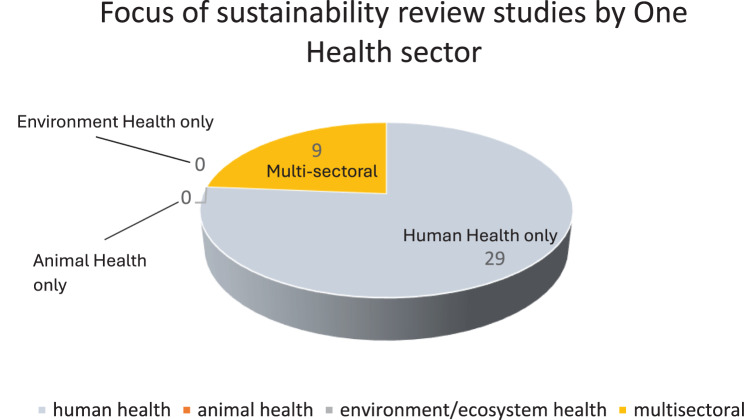
Pie chart showing breakdown of sustainability focused health studies by sector: animal only/environment only/human only/multisectoral

Overall, human health predominates as a central theme with 29/38 (76%) of review studies on sustainability exclusively limited to the sector. Sub-categories of enquiry around sustainability within the human health sector were categorized most often as continued implementation of programmes, interventions, services, strategies and/or systems. The remaining 9/38 (24%) of review studies identified were multisectoral in character (see Fig. 3) [16, 18, 31, 39, 43, 44, 53, 56, 57]. Of these, 6/9 (67%) included two sectors namely human health and environment/ecosystem health, with a focus on both sustainability as a process as well as an outcome. 2/9 (22%) involved all three sectors with the Perry et al. study of 2018 making specific reference to One Health, and the earlier review study of Du Plessis and Brandon framed around social-ecological systems [18, 56]. Only 1/9 (11%) multisectoral review studies was focused on animal and ecosystem health together [53] and notably there were no (0%) review studies identified dedicated exclusively to animal health or ecosystem health on their own.

The AMSTAR II quality appraisal of the 38 included studies is presented in online appendix 3. Overall, the methodological rigour and quality of review studies has improved over the years with many of the systematic reviews published since 2017 scoring very well on the AMSTAR II criteria (e.g. by establishing methods and publishing protocols prior to the review). In general, human health focused sustainability review studies were of higher methodological quality and more systematic in their approach than those that were multisectoral or more focused on animal or environmental health outcomes. With many of the systematic review studies identified either conceptual or scoping in nature, some of the AMSTAR II criteria were not applicable during analysis e.g. those criteria relating to a quantitative meta-analysis.

### Definitions of sustainability used across review studies

For multisectoral review studies, 3 out of 9 (33%) did not use a previously agreed and published definition for sustainability and developed their own definition for the purpose of their particular study [44, 53, 56]. This included the only review study identified that excluded human health and focused on the health of animals (mariculture in this case) and the environment [53]. The remaining 6 (66%) multisectoral review studies all adopted the Brundtland Commission definition of sustainability, with 2 (33%) of these studies also utilising a more focused definition related to their specific topic of enquiry (i.e. sustainability as related to food and nutrition security and sustainable diets respectively) [16, 18, 31, 39, 43, 57]. Irrespective of which definition was used, all multisectoral review studies however, did frame [29, 55] their analysis around the Brundtland Commission pillars of sustainability, i.e. society (including health), economy and environment.

For the 29 of 38 (76%) review studies focused solely on the human health sector, only 3 (10%) did not use a previously developed or published definition of sustainability. For those that did, the most commonly cited definitions for sustainability were those developed by Shediac-Rizkallah and Bone (1998), Scheirer (2005), Stirman et al. (2012), and Moore et al. (2017). Interestingly, only 2/29 (7%) health sector focused review studies chose to adopt and frame their analysis around the Brundtland Commission definition of sustainability and its pillars of environment, economy and society [29, 55].

### Determinants of sustainability and its characteristics across sectors

As stated previously, from our detailed search no review studies were identified that focused solely on animal or environmental health. This suggests that human health is almost always a consideration or factor in analyses around sustainability for the environmental and animal sectors. Almost all review studies identified with a focus on environmental health also considered human health (8/9), and as only (3/9) paid specific attention to animal health (two of these involved all three sectors and one included only the environmental and animal health sectors), we conducted a broader comparison of determinants and characteristics of sustainability between multisectoral review studies (9/38), and those that were human health focused only (29/38). Table 1 summarises these findings following a deductive thematic analysis. As the included reviews used heterogeneous methods and reported differing levels of detail, we did not present determinants by frequency, nor is equal tabular space intended to imply equal empirical weight. Several characteristics appear across multiple domains (e.g., capacity building) because they were described by authors as operating at more than one system level; this reflects how the literature conceptualises mutually reinforcing rather than discrete categories. Emerging determinants or characteristics of sustainability were categorized across process-oriented themes of the ‘how’, ‘when’, ‘who/by whom’ to sustain and the more outcome-oriented ‘why’ and ‘what’ areas to sustain, while recognizing that these factors, barriers and enablers exist as a continuum across processes and outcomes and can sometimes overlap [23, 36]. Our findings indicate, that as the academic literature around sustainability has advanced through the years, human health sector review studies have become relatively more precise and detailed in their elaboration of process-related factors of sustainability around focused sets of outcomes. Multisectoral review studies, on the other hand, have in general, tended to address a more diverse set of holistic outcomes across sectors often framed around particular issues of topical interest (e.g. sustainability of healthcare services and operations, sustainable food systems and health, or green infrastructure across urban and rural developments schemes and its links to health etc) [18, 43, 44, 57].

**Table 1:** A comparison of determinants and characteristics of sustainability identified in human health sector focused review studies versus multisectoral health focused review studies

Review studies (*N* = 38)	Process-focused determinants/characteristics of sustainability	Outcome themes and characteristics of sustainability
Multisectoral (*N* = 9)	1. **Community Participation:** Active engagement of communities in sustainability efforts.	1. **Social Equity:** Achieving social equity and inclusion in sustainability initiatives including attention to vulnerable groups and gender-based inequalities.
	2. **Education and Awareness:** Raising awareness and providing education on sustainability issues.	2. **Health Dimensions:** Integrating health considerations into societal well-being, including public health impacts and disease control.
	3. **Politics, Policy and Governance:** Influence of politics, policies and governance structures in promoting sustainability.	3. **One Health Outcomes:** Integrating human, animal, and environmental health for holistic sustainability impact assessment.
	4. **Monitoring and Evaluation:** Systematic tracking of program progress and outcomes.	4. **Biodiversity:** Maintaining and enhancing biodiversity as part of environmental sustainability.
	5. **Partnership Development:** Establishing and nurturing collaborative relationships.	5. **Environmental Restoration:** Initiatives aimed at restoring degraded environments and promoting ecosystem health.
	6. **Adaptation Strategies:** Flexibility to respond to changing environmental and social conditions.	6. **Integrated Ecosystem Management:** Holistic management practices that consider the interdependence of human, animal, and environmental health.
	7. **Capacity Strengthening:** Enhancing capabilities at individual, organizational, and community levels.	7. **Natural Resource Management:** Sustainable management of natural resources to prevent degradation and ensure equitable access to water, air, soil, land across living species.
	8. **Inclusive Policymaking:** Ensuring diverse voices are represented in policy decisions.	8. **Environmental Impact:** Addressing environmental sustainability through improved recyclable/non-recyclable resource management and waste/pollution control in built and natural environments.
	9. **Knowledge Exchange:** Platforms for sharing best practices and lessons learned.	9. **Climate Change:** Mitigation and adaptation strategies to address climate change impacts.
	10. **Strategic Planning:** Long-term planning to guide sustainability efforts.	10. **Economic/Financing Access:** Ensuring economic viability and access to financial and other resources.
	11. **Resilience Planning:** Preparing for environmental and socio-economic disruptions and health emergencies.	11. **Broader economic factors:** Micro-/mesoeconomic efficiency, macroeconomic stability
	12. **Systems Thinking:** Approaching sustainability as interconnected systems across sectors.	12. **Sustainable Livelihoods:** Supporting sustainable livelihoods, particularly in vulnerable communities.
	13. **Ethical Practices:** Upholding ethical standards and values in programme processes.	13. **Food and Nutrition Security:** Ensuring food availability, accessibility, and sustainability in food systems.
	14. **Corporate Social Responsibility:** The role of corporate behavior and ethical practices in sustainability.	14. **Resilience:** Building resilience in systems and communities to adapt to changes and shocks including health emergencies.
		15. **Cultural Preservation:** Protecting and promoting cultural heritage and diversity including indigenous knowledge systems and historically marginalized groups.
		16. **Wildlife Conservation:** Protecting wildlife populations and their habitats as part of sustainable ecosystems.
		17. **Livestock Health:** Improving livestock health and well-being to enhanced food security and economic stability.
		18. **Cross-Sectoral Resilience:** Strengthening resilience through collaboration across sectors and disciplines.
Human health focused (*N* = 29)	1. **Governance and Leadership:** Emphasis on good governance, strong leadership, programme/service champions and strategic planning to ensure sustainability.	1. **Public Health/health service impact:** In terms of reduced morbidity/mortality and improvements in health outcomes/well-being in different target groups over time.
	2. **Organizational Culture:** The role of organizational culture in fostering sustainability through values, norms, and practices.	2. **Evidence-Based Impact:** Outcomes supported by strong data and research.
	3. **Upholding the rule of law:** Adhering to principles of justice, human rights, transparency, combating corruption.	3. **Knowledge Transfer:** Effective dissemination of knowledge across sectors and communities.
	4. **Resource Management:** Efficient allocation and management of resources.	4. **Empowerment:** Empowering communities and individuals to take ownership of sustainability efforts.
	5. **Partnerships and Collaboration:** Importance of multidisciplinary partnerships and stakeholder engagement.	5. **Equity and Inclusion:** Ensuring equitable access to benefits across diverse groups including vulnerable groups and gender dimensions.
	6. **Policy Change:** Influencing legislation and regulations to support sustainability.	6. **Behavioral Change:** Sustained positive behavior changes among beneficiaries.
	7. **Funding Stability:** Reliable and diversified funding sources are crucial for long-term sustainability.	7. **Program Sustainability:** Ensuring programs continue to deliver benefits over time.
	8. **Capacity Building:** Continuous training, education, and skill development to enhance organizational and community capacity.	8. **Organisational/Programme resilience building:** Enhancing the capacity to withstand and recover from challenges and emergencies.
	9. **Communication:** Effective communication channels within and outside the organization to promote transparency and engagement.	9. **Community Resilience:** Building local community-wide capacities to manage risks, emergencies and adapt to changes.
	10. **Adaptability/fidelity:** Programmes’ ability to adapt to changing contexts and integrate new knowledge while maintaining fidelity/quality assurance.	
	11. **User Involvement:** Active involvement of users and beneficiaries in program design and implementation.	
	12. **Program Evaluation:** Ongoing monitoring and evaluation to measure effectiveness and inform improvements.	
	13. **Technical Support:** Support from technical experts and top management attention to sustain initiatives.	
	14. **Accountability:** Clear roles, responsibilities, and accountability mechanisms.	
	15. **Innovation and Implementation:** Focus on innovative practices and effective implementation strategies.	
	16. **Decision-Making Processes:** Structured approaches for inclusive and evidence-based decision-making.	
	17. **Stakeholder Engagement:** Mechanisms to engage diverse stakeholders consistently.	
	18. **Knowledge Management:** Systems for capturing, sharing, and utilizing knowledge effectively.	
	19. **Cross-Sectoral Integration:** Mechanisms to facilitate integration across different sectors and disciplines.	
	20. **Interdisciplinary Collaboration:** Encouraging interdisciplinary teamwork to address complex sustainability challenges.	
	21. **Community-Based Approaches:** Engaging local communities as key stakeholders in the process.	
	22. **Feedback Mechanisms:** Establishing continuous feedback loops to refine strategies.	
	23. **Operational Efficiency:** Streamlining processes to maximize resource utilization.	
	24. **Capacity for Innovation:** Building an environment conducive to experimentation and learning.	
	25. **Process Optimization:** Regular review and optimization of workflows.	
	26. **Change Management:** Strategies to manage transitions and organizational changes.	
	27. **Institutional Alignment:** Ensuring consistency between programme goals and institutional missions.	
	28. **Data-Driven Decision Making:** Using data analytics to guide strategic planning and operations.	
	29. **Multi-Stakeholder Platforms:** Creating platforms for collaboration among various stakeholders.	
	30. **Policy Advocacy:** Engaging in advocacy to influence policy changes supportive of sustainability.	
	31. **Monitoring Systems:** Advanced systems for tracking progress and outcomes.	
	32. **Regulatory Compliance:** Ensuring alignment with relevant regulations and standards.	
	33. **Sustainability Planning:** Incorporating sustainability considerations into all stages of planning.	
	34. **Resource Mobilization:** Effective strategies for securing financial and human resources.	
	35. **Strategic Partnerships:** Building alliances that leverage complementary strengths.	
	36. **Organizational Learning:** Fostering a culture of continuous learning and improvement.	
	37. **Equity in Processes:** Ensuring fairness and inclusiveness in process design and implementation.	
	38. **Ethical Governance:** Upholding ethical standards in decision-making and governance structures.	

Multisectoral studies in our analysis (9/38), always characterized sustainability in terms of environmental, social and economic viability with complex, system based and adaptive approaches to health and well-being across sectors. By design, human health sector review studies (29/38) rarely considered factors outside of delivery of a health sector related function or process, and its related human health outcomes, and co-benefits or trade-offs across sectors were not a feature of their analyses. While individual sustainability assessments in the human health sector have routinely used focused linear or mechanistic approaches around narrow parameters, the systematic review literature in our study recognizes that while these approaches might be easier and technically less complex to carry out, sustainability as a concept has increasingly over time come to be characterized as a complex ecosystem of time-bound and interrelated factors across sectors, scales, determinants and relationships between stakeholders and their environment. A summary of thematic areas of similarity and difference between human health sector focused reviews and multisectoral review studies is presented in Table 2 below. To avoid implying uniform consensus, Table 2 distinguishes themes that were widely shared across sectors from those (e.g., technology advancement) that arose more selectively or context-dependently. For these sector-specific themes we include clarifying notes on why they matter for sustainability outcomes, based on the rationale provided in the underlying reviews.

**Table 2:** Thematic similarities and differences in characteristics and conceptual approaches to sustainability between human health sector and multisectoral health focused review studies

Thematic similarities	Thematic differences
1. Emphasis on ethical and effective **Governance and Leadership** as critical drivers of sustainability in both datasets.	1. **Timeframes for sustainability:** • **Human health:** More precise in presenting timeframes including at initiation, implementation and post-implementation. Timeframes vary from between 6 months post-initiation/post initial funding to several years(1–16 years) thereafter with interim monitoring of progress. • **Multisectoral health:** Did not usually make explicit mention of timeframes, however when they are mentioned tend to be medium to long-term timeframes for environmental, economic and social outcomes relating to health that can extend inter-generationally.
2. Focus on **Partnerships and Collaboration** to leverage multidisciplinary expertise for sustainability outcomes.	2. **Funding Environment:** • **Human health:** Emphasize funding stability and diversified financial streams within organizational contexts. • **Multisectoral health:** Focus more on external factors like strategic funding related to environmental sustainability projects and resilience funding.
3. Importance of **Capacity Building** and continuous skill development to strengthen sustainability efforts.	3. **Economic Factors:** • **Human:** Economic considerations are tied to resource mobilization and efficiency within organizations. • **Multisectoral health:** Highlight broader economic themes like economic access, sustainable livelihoods, and market resilience linked to environmental and community sustainability. Animal and livestock health sectors place a particularly strong emphasis on economic viability and livelihoods in relation to sustainability.
4. Need for strong **Policy and Governance** structures to create enabling environments for sustainability.	4. **Workforce/Employees:** • **Human health:** Strong focus on workforce development, organizational roles, and leadership capacity within structured entities. • **Multisectoral health:** Less emphasis on structured workforce roles, with more focus on community-based capacities and local leadership for sustainability.
5. Role of **Monitoring and Evaluation** in tracking progress and assessing sustainability impacts.	5. **Technology Advancement:** • **Human health:** Incorporate technology as part of data-driven decision-making and knowledge management systems. • **Multisectoral health:** Emphasize technology utilization for environmental monitoring, sustainable agriculture, and renewable energy solutions.
6. Significance of **Community Participation** to ensure interventions are contextually relevant and accepted.	6. **Socio-Political Environment:** • **Human health:** Address the political environment in terms of policy advocacy and creating enabling governance structures. • **Multisectoral health:** Highlight the socio-political context related to environmental justice, climate change activism, and global sustainability agendas.
7. Focus on **Adaptation Strategies** to address dynamic environmental and organizational changes.	7. **Leadership and Ethics/Values:** • **Human health:** Focus heavily on ethical governance, leadership within organizational hierarchies, and institutional alignment. • **Multisectoral:** Ethics are reflected through values tied to environmental stewardship, equity, and community-driven leadership models.
8. Emphasis on **Strategic Planning** to guide long-term sustainability objectives.	8. **Evidence-Based Approaches:** • **Human health:** Strong emphasis on evidence-based impact, program evaluation, and data-driven planning within organizational settings. • **Multisectoral:** Evidence-based approaches are more integrated into environmental monitoring, biodiversity assessment, environment and social/health sustainability impact evaluations.
9. Use of **Knowledge Exchange** mechanisms to share best practices and lessons learned.	9. **Cross-Sectoral Integration:** • **Human health:** Address cross-sectoral integration primarily through multi-stakeholder platforms within governance systems. • **Multisectoral health:** Broader focus on One Health and ecosystem-based management approaches that inherently require cross-sectoral collaboration.
10. Commitment to **Resilience Building** in communities and systems to withstand shocks.	10. **Resilience Focus:** • **Human health:** Resilience is framed around organizational adaptability and program sustainability. • **Multisectoral health:** Broader focus on ecosystem resilience, climate adaptation, and community-based resilience.
11. Consideration of **Environmental Impact** as a key component of sustainability strategies.	11. **Knowledge Systems:** • **Human health:** Focus on structured knowledge management systems within institutions. • **Multisectoral health:** Emphasize indigenous knowledge, community-based learning, and knowledge exchange across sectors.
12. Inclusion of **Equity and Inclusion** as cross-cutting principles to promote fairness in access to resources, opportunities and outcomes.	12. **Program Sustainability:** • **Human health:** Framed in terms of program longevity, strategic planning, and institutional learning. • **Multisectoral health:** Linked to environmental conservation, biodiversity protection, and sustainability in natural resource management.
13. Promotion of **Ethical Practices, transparency and accountability** across governance and implementation processes to build trust with stakeholders.	13. **Climate Change:** • **Human health:** Limited focus, often indirect through resilience strategies. • **Multisectoral health:** Explicit focus on climate change mitigation and adaptation as core sustainability outcomes.
14. Importance of **Resource Management** for efficient allocation and use of both financial and human resources.	14. **Health Focus:** • **Human health:** Focus on public health outcomes within programmatic contexts. • **Multisectoral health:** Broader health outcomes including One Health relevant disease control and well-being, and environmental determinants of health.
15. Shared reliance on **Evidence-Based Approaches** to inform decision-making and enhance sustainability outcomes.	15. **Cultural Dimensions:** • **Human health:** Less emphasis on cultural factors in sustainability. • **Multisectoral health:** Explicit outcomes related to cultural preservation, traditional knowledge, and community heritage.
16. Promotion **of Cross-Sectoral Collaboration and Solidarity** across both datasets.	16. **Environmental Outcomes:** • **Human health:** Address environmental factors indirectly through policy and organizational sustainability. • **Multisectoral health:** Direct focus on ethics and stewardship through environmental restoration, wildlife conservation, and ecosystem management.
17. Integration of **Sustainability as a core value** prioritizing long-term impacts.	17. **Organizational culture, processes and systems thinking:** • **Human health:** Generally, more linear approaches focusing on organizational systems and performance metrics, emphasizing data-driven planning, optimization, change management, institutional alignment, organizational ethics, professional codes of conduct and regulatory compliance. • **Multisectoral health**: Rooted in systems thinking, recognizing the interconnectedness of ecological, social, and economic systems. Emphasize environmental and ecological processes, integrated ecosystem management, community empowerment, cultural preservation and indigenous/local perspectives.
	18. **Wider considerations beyond health across society, economy and environment:** • **Human health:** Limited consideration of wider factors outside of human health. • **Multisectoral health:** Consider co-benefits and trade-offs more often. Include specific references to Climate Change mitigation and adaptation, sustainable livelihoods and food security outcomes, wildlife conservation, ecosystem services and improved waste/pollution and natural resource management.
	19. **Human-Animal-Environment (One Health) and intergenerational equity:** • **Human health:** Lack explicit reference to integrated ethical frameworks considering health across sectors, species and generations, focusing more on current programmatic sustainability and linked health outcomes. • **Multisectoral health:** Ethical frameworks explicitly include One Health principles, valuing the inter-connectedness of human, animal, and environmental health. More often consider the rights of future generations and the long-term health of the planet.
	20. **Social Justice and Advocacy:** • Human health: Focus more on organizational integrity and professional ethics rather than broader social advocacy. • Multisectoral health: Strong emphasis on social justice ethics, environmental activism, and advocacy for marginalized communities.

With only a third (3/9) of sustainability focused multisectoral review studies considering the animal health sector, the concept is relatively less well defined and characterized therein. While all multisectoral studies (9/38 in our analysis) have adopted the conceputalisation of sustainable development, for the animal health sector in particular, the concept boundaries are not clearly delineated with some overlap around conceptual approaches to food security and food systems [18]. For the environmental health sector (6/9 multisectoral review studies in our analysis) there is the longstanding Brundtland definition around the concept and a clear set of outcomes in the literature. However, a precise characterization around boundaries and determinants or antecedents is less well developed in the six studies identified through our analysis. For the human health sector there has been steady progression in the maturation of sustainability as a concept, with a growing body of academic literature of increasingly high methodological quality helping characterize both process and outcomes at a variety of scales and levels of organization of the sector. Definitions for sustainability in the human health sector have also continued to evolve with increasing consensus around particular characteristics becoming the norm [23, 28–30, 32, 36, 63]. However, in relation to the academic literature around health emergencies and disasters in particular, there is a lack of conceptual clarity and clear boundaries between the related ideas of sustainability and resilience – a finding we noted across all three sectors. In dealing with outbreak or health emergency preparedness, response and recovery there appears to be an academic preference in the literature to use the term resilience, albeit with many of the characteristics and determinants being similar [18, 43, 80, 81]. From our examination of the published literature and using Morse et al. pragmatic concept analysis approach (see open-access protocol paper) we broadly summarized the relative maturity of the concept of sustainability across each the environmental, animal and human health sectors in Table 3 below. The pragmatic utility method, using literature as data, examines concept maturation through a number of criteria such as the definition of the concept (clarity and consistency), the characteristics of the concept, the conceptual preconditions and outcomes, and the conceptual boundaries [82, 83]. These indicators were applied descriptively and not for ranking sectors; rather, they highlight areas where conceptual development is more advanced or where additional methodological work could strengthen research capacity.

**Table 3:** Relative conceptual maturity of sustainability across the human, animal and environmental health sectors based on identified review studies (*N* = 38)

	Definitions	Attributes/characteristics	Preconditions/determinants	Consequences/outcomes	Boundaries	Relative Maturity
Environmental health	+++ Longstanding, widely accepted definition (Brundtland et al.)	++	+	+++	**+**	++
Animal health	+ + Uses Brundtland et al. definition with some overlap with food security/food systems definitions	+	Least well defined	++	**+**	Least mature +
Human health	++ A number of operational definitions in use	++	+++	++	**++**	+++

### Additional review analysis - sustainability as a primary outcome measure in an evaluation

Of the 22 review studies (see appendix 2) where sustainability was a primary outcome measure of an evaluation, 17 had a focus solely on human health, one focused exclusively on animal health and four had a multisectoral focus involving human and environmental health. A range of frameworks and tools were used across the studies to evaluate sustainability with most studies using an already existing framework or a combination of existing frameworks to structure their analysis. These would often be drawn from existing sustainability or implementation research literature. For the animal health focused and multisectoral focused review studies, all of them adopted the Brundtland definition and UN SDG conceptualization of sustainability around the three pillars of environment, economics and society [12]. For the 17 review studies focused on human health, the Moore et al. 2017 definition was most often adopted [17] with other published definitions and conceptualisations also being cited, albeit less frequently. 7 of the 22 studies did not formally adopt a previously published definition or conceptualisation of sustainability and defined or conceptualised it themselves for the purposes of their respective evaluation reviews. Only 7/22 studies made specific mention of a timeframe over which sustainability was evaluated with periods ranging from 6 months post-initiation to 10 years. Thuo et al. for example, who evaluated sustainability of Neglected Tropical Disease programmes, proposed assessments over three phases; a scale up, maintenance (over 5–10 years) and a scale down phase where disease control or elimination had been achieved [74]. Several of these studies made reference to a one- or two-year time period following completion of initial implementation or following a period where external funding support had been withdrawn. None of the multisectoral or animal health focused studies made any specific mention of a timeframe over which to evaluate sustainability further suggesting a lack of consensus, consistency of approach, boundaries and maturity around the concept of sustainability in these sectors. The limited explicit temporal framing across sectors has important implications: it complicates comparability between studies, obscures the time horizons required for system-level change, and hinders alignment between programme cycles and sustainability goals. These implications are now further elaborated in the Discussion. The summary details of each sustainability framework or tool identified for these evaluation reviews can be found in appendix 2.

## Discussion

Our umbrella systematic review is the first of its kind to examine the concept of sustainability across the One Health relevant sectors of human, animal and environment/ecosystem health. The concept of sustainability and its evaluation from our analysis is most well developed academically in the published review literature in the human health sector. Most review studies of conceptual and analytical approaches to sustainability (76%) as well as those relating to evaluating sustainability as a primary outcome measure were limited to the human health sector. Multisectoral review studies (24%) almost always involved at least the human health sector and usually the environment sectors but very rarely the animal health sector. This suggests a relative lack of maturity around the concept and its application in the animal sector in particular. Multisectoral review studies and those relating specifically to animal or environment/ecosystem health all predicate their conceptual understanding and analysis on the Brundtland Commission work that both defined sustainable development and structured its outcomes around the three pillars of society, economy and environment. Although our synthesis uses the three-pillar framing because it was consistently applied in the included reviews and the most widely adopted, we acknowledge that sustainability scholarship has proposed additional or alternative pillars—such as culture, politics, or governance—which are important for fuller conceptualisation. These are compatible with our findings, which also embed equity, justice, participation and socio-ecological context. For sustainability from a process driven perspective, the majority of studies have progressively built on the early work of Shediac-Rizkallah and Bone and have sought to refine and adapt that framework to different human health contexts. Following, publication of the Moore et al. definition for sustainability in human healthcare contexts, review studies have increasingly chosen to either adopt this definition fully or use it as starting point for their own particular analytical needs [17, 54].

### A meta-conceptual framework for sustainability across human, animal and environmental health

**Fig. 4:**
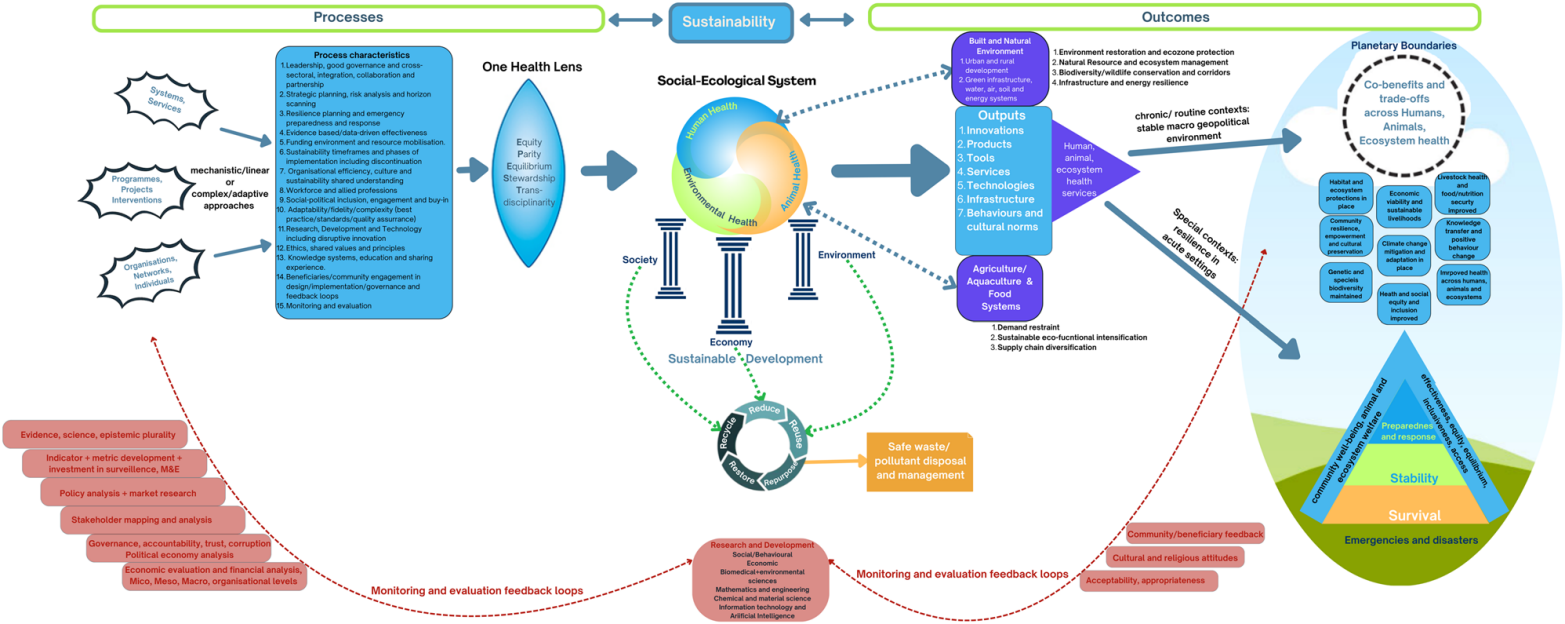
Meta-conceptual framework for sustainability across the one health paradigm

Our deductive thematic analysis has identified a wide range of determinants and characteristics of sustainability as well as conceptual frameworks for assessing them. While our analysis did highlight varying characteristics and determinants of sustainability between human health sector only and multisectoral review studies, there were numerous conceptual similarities and overlaps. Where differences did exist these were largely related to emphasis, or the relative importance of a characteristic or determinant rather than a contradictory or opposing factor. For multisectoral review studies, additional factors, particularly, those concerning animal health or ecosystem health outcomes, as well as co-benefits and trade-offs relating to wider societal conditions, the economy and the environment (including climate change) were important considerations when characterizing sustainability. With the One Health approach also maturing conceptually in recent years, combined with the publication of a widely accepted definition, a theory of change for its operationalization, and a global action plan to support implementation, integrating the concepts has become increasingly important. Fig. 4 and its accompanying description in Box 2, represents a meta-conceptual framework for sustainability to support analysis across the One Health paradigm, bridging human, animal and ecosystem health at different levels. These range from the process-driven practitioner’s or implementer’s perspective on sustainability, to the global SDGs-driven outcomes of interest and impact around society, economy and environment [28, 29, 31]. Although Fig. 4 derives from empirical themes from the synthesis, it necessarily retains some normative orientation because the literature itself presents sustainability both as an analytical construct and as an aspirational systems goal. This conceptual synthesis maps what is most explicitly present in existing review literature as evidence derived but does not quantify strength. Based on our findings and the current literature around One Health principles and operationalisation, the meta-conceptual framework serves as an evidence-based normative guide that is adaptable across scales, levels and a wide spectrum of multisectoral One Health issues to assess and structure evaluations of sustainability. From our wider reading of the literature and our tabulated results (Tables 1 and 2) we aggregated process-focused characteristics identified in our review into a list of 15 factors that were integrated into the conceptual framework. For the workforce for example, professional standards and personal development, career pathways and progression, acknowledgement of value and worth, and positive working environments along with an organisation wide shared understanding of sustainability, were all key determinants of sustainability [28, 46, 47]. Aggregated outcome-related themes were similarly represented on the process-outcome continuum of the framework (Box 2).

**Box 2:** Description of Meta-Conceptual Framework for Sustainability

This framework presents a time-bound systematic approach to integrating processes, sustainability determinants, and outcomes within a socio-ecological system. The figure follows the progression of sustainability from initial implementation to long-term impact, guiding organisations, interventions, services, and systems through a structured evaluation and adaptation process.
**1. Entering the Framework: Sustainability as a Process (Left Side)**
Sustainability begins with structured processes that define and guide health, environmental, and economic interventions. These processes are divided into three tiers of implementation:
• Systems & Services: Large-scale, institutional efforts (e.g., health systems, environmental policies). • Programmes & Projects: Targeted initiatives that support sustainability goals (e.g., vaccination programs, conservation projects). • Organisations, Networks, & Individuals: The people and communities driving change.
At this stage, sustainability efforts select choose between mechanistic (linear) and complex/adaptive approaches, influencing how flexibility, governance, and integration are applied. A set of 15 process characteristics ensures sustainability is built on strong governance, funding stability, strategic planning, research, equity, and community/beneficiary participation. These factors create the foundation for sustainable One Health initiatives.
**2. The One Health Lens: Moving into a Systems-Based Perspective (Centre)**
As we progress through the framework, initiatives are filtered through a One Health Lens, ensuring sustainability is approached holistically applying core principles of:
• Equity (fair distribution of benefits across sectors, scales and communities). • Parity (inclusiveness in decision-making/priority setting and opportunities with appropriate focus on marginalised and vulnerable groups). • Equilibrium (maintaining a harmonious balance within ecosystems and across societies with a respect for biodiversity, animal welfare and the intrinsic value of all living things). • Stewardship (responsible resource management and the responsibility of humans to change behaviour and adopt sustainable solutions). • Transdisciplinarity (integrating knowledge across disciplines and respect for different epistemic traditions across sectors and societies).
This approach feeds into the Social-Ecological System, represented as an inter-linked triad of human, animal, and environmental health embedded within society, economy, and the environment (visually represented by the three pillars of sustainable development, reinforcing the Brundtland Commission’s conceptualisation of sustainability). This phase transitions sustainability efforts into structured outputs that shape outcomes and long-term impact:
• Innovations, tools, and technologies for sustainable development. • Infrastructure & green urban/rural planning to improve and protect built and natural environments with a focus on enhancing resilience. • Sustainable food (agriculture/aquaculture) systems that ensure long-term ecological balance alongside food/nutrition security. • Waste & pollutant management to reduce environmental harm and promote clean water, air, soil and energy.
**3. Achieving Sustainability Outcomes (Right Side)**
At this stage, sustainability efforts at the macro level, influence societal, economic, and environmental development. Outcomes are shaped by two contextual settings:
• Chronic/Routine Contexts: Sustainability focused initiatives in a predictable, slower moving environment. • Special Contexts: Resilience-building efforts in crisis settings (e.g., pandemics, natural disasters, conflict etc).
Sustainability outcomes are closely tied to planetary boundaries, emphasising the importance of: • Climate change mitigation & adaptation. • Biodiversity conservation & habitat protection. • Food & nutrition security. • Equitable access to health and economic resources.
A hierarchical resilience framework (modelled on Maslow’s hierarchy) illustrates sustainability across different levels of stability: 1. Survival (Emergency Response): Immediate crisis management (e.g., pandemic response, disaster relief). 2. Stability: Ensuring continued function and recovery after the initial response. 3. Preparedness & Response: Developing mechanisms and capacity for addressing future crises.
This trajectory ensures sustainability efforts are continuously evolving, allowing societies, ecosystems, and economies to adapt without compromising future generations.
**4. Closing the Loop: Monitoring, Evaluation, and Adaptation (Bottom Section)**
Sustainability is not static—it requires continuous feedback, monitoring, and adaptation. The bottom section of the framework represents a self-sustaining evaluation cycle, ensuring that lessons learned are fed back into the system through: • Scientific research, evidence, and knowledge-sharing. • Stakeholder mapping, governance accountability, and policy analysis. • Economic & financial assessments to ensure viability. • Community & cultural feedback mechanisms for social acceptability/appropriateness and to guide priority setting. This cycle strengthens adaptive capacity, making sustainability initiatives more resilient, responsive, and effective over time.

In the framework, process related factors integral to the design and delivery of multisectoral systems, strategies, services and other initiatives for health are viewed through a One Health lens in line with its principles and ways of practice. These include adhering to values of equity between sectors and communities, sociopolitical parity (inclusiveness of all segments of society including marginalized and vulnerable groups), socioecological equilibrium between species and ecosystems, human stewardship of the natural world and its resources, and a commitment to transdisciplinarity, and respect across different epistemic traditions [31, 55, 56]. These principles are well aligned to our reading of the sustainability for health literature where the inclusion of indigenous communities and other marginalized groups, equitable access and outcomes, animal welfare, balanced and thriving ecosystems, and the stewardship role of human communities are all emphasized. Risks, trade-offs and potential benefits across human, animal and ecosystem heath are then examined across the whole of the Social Ecological System (SES) in line with evidence-based best practice, the latest science and technology, and all relevant disciplines and sources of knowledge [31, 56]. It is important to note here that while the literature we reviewed in our results often used ‘interdisciplinary’ and ‘transdisciplinary’ interchangeably these are well-defined terms in the broader academic literature. Our framework is compatible with the International Science Council’s distinction, recognising that transdisciplinarity involves co-creation of knowledge with actors beyond academia, which is central to One Health sustainability efforts [84–86].

Outputs, services and products developed to support short-, medium- and long-term outcomes that have a positive impact on health and well-being across human societies, animals and our ecosystems have then to be considered across all pillars of sustainable development, i.e. in terms of their social, economic and environmental implications. This phase involves several different considerations and types of analyses. From our examination of the literature these include: 1)lifecycle analysis for products, pollutants and pathogens (3 Ps), 2) environmental impact assessments (with inclusion of health and social impact assessment), 3) reduce-recycle-reuse-repurpose-restore initiatives while ensuring safe waste/pollutant disposal systems for non-recyclable materials, 4) regenerative agriculture and sustainable eco-functional intensification coupled with demand restraint and supply chain diversification for food systems and agricultural products, and 5) economic assessments that account for micro/mesoeconomic efficiency and macroeconomic stability while supporting livelihoods, financial viability and sufficiently examining positive and negative externalities (trade-offs) [43–45, 54, 58]. For the individual entrepreneur, service or enterprise however, a narrower financial analysis predicated on production and marketing costs and profits from sales is often of more interest than the broad accounting frameworks of economic analysis which also measure the social and environmental costs/benefits on society [53]. These types of analyses and considerations apply in all areas of development where human activities have impact, be they urban, rural, built or natural environments.

On the outcomes and impact end of the sustainability framework we developed two broad categories. The first category focuses on the sustainability and resilience needed for navigating acute events such as health emergencies (e.g. from an outbreak be it in human or animal populations to the sudden loss of a critical health service) or disasters, whether they are natural or man-made. Borrowing from psychological theory in the form of Maslow’s pyramid and the hierarchy of needs, the base layer represents human and ecosystem needs for sustainability around simple survival, the next level up focuses on stability and recovery from the emergency, and the highest level prioritises building future preparedness and response capacity when resources allow [87]. The second category focused on sustainability in the context of challenges of a more chronic nature or ever-present concern, illustrated by planetary boundaries in danger of being breached (e.g. ranging from routine maintenance of a health service, to macro threats to geophysical boundaries such as anthropogenic-induced climate change (greenhouse gases) and ecosystem damage, growing/ageing populations and species competing for finite resources and space) [88]. Multiple bi-directional feedback loops between the intended beneficiaries and local communities, to those designing, governing and implementing strategies (of which communities should be a key part), and the scientists and researchers developing and innovating around the technology, behaviour change and evidence base for the most effective interventions are a key feature of the framework. Trust in in government and across society, particularly after the COVID-19 Pandemic, and measures to combat corruption are also increasingly recognized as key components to monitor for long-term sustainability [29, 58]. Sufficient resourcing and use of the appropriate monitoring and evaluation (M &E) tools to support each phase or element of the framework are themselves an enabling determinant of sustainability, and ensure feedback loops are effective. These M&E tools include regular political economy analysis, stakeholder mapping and analysis, horizon scanning around new science and emerging risks, disruptive technologies (such as artificial intelligence or blockchain), economic analysis and financial planning, and integrating an evolving evidence-base through regular knowledge exchange between sectors, scientists, policy makers and communities.

### A new integrated definition for sustainability across the one health paradigm

From our reading of the literature, there has been a steady evolution in approaches to sustainability with models that adopt a linear or mechanistic approach increasingly giving way to models which recognize and embrace system complexity and recognize the inter-relatedness of external contextual factors to internal processes around health-related initiatives or interventions. While more mechanistic approaches have a role to play in the analysis of specific project or intervention delivery, or for evaluating sub-components of health related services/systems, there is a growing consensus that analytical approaches to sustainability have to be able to account not just for fidelity, i.e. the quality assured delivery of an intervention or service as originally intended, but also adaptability as circumstances, understanding, socio-political context, health needs and knowledge systems change over time. This can include when appropriate, sunset clauses to discontinue services or interventions when outcomes or intended impacts have been achieved [23]. One of the most important examples globally of this need for transition planning and discontinuation of services, is the global Polio control programme and its associated workforce [89]. As the world inches closer to polio elimination, transitioning the hundreds of thousands of healthcare workers that have helped deliver this key success has been a significant challenge in numerous countries and continues to be in the few jurisdictions where polio is still endemic or at risk of re-emergence. On process-focused approaches to sustainability, our analysis as well as existing analysis around One Health shows that the animal health and environmental health sectors are relatively less well developed and in need of greater investment if we are to have equity between sectors. There is a well-described inequity in power and resourcing dynamics between the human, animal and environmental health sectors and for sustainability to be ensured across the One Health paradigm, equity itself as an outcome, requires resourcing and investment [6, 19, 56]. Several of our included reviews and the wider literature highlight implicit power imbalances shaped by funding flows, institutional mandates and the predominance of human-health epistemologies in defining multisectoral priorities. Our integrated framework therefore embeds governance, participation and equity explicitly to address these asymmetries and ensure that sustainability is not conceptualised solely from the vantage point of dominant sectors [4, 6, 18, 56]. While our framework differs from implementation-science models such as the Consolidated Framework for Implementation Research (CFIR) or RE-AIM for example [59, 72], it complements them by focusing on intersectoral characteristics of sustainability across ecological, animal health, social and institutional systems—areas where sector-specific models tend to be limited and where ours allows One Health practitioners to tailor their analysis and evaluations of sustainability to particular contexts.

By examining the fullness of the systematic review literature around sustainability across human, animal and ecosystem health as well as conceptual approaches to One Health and the SDGs, we propose a new integrated definition for sustainability that complements our meta-conceptual framework. This definition of sustainability builds on existing conceptualisations and previously used definitions, and reflects the need to balance process-driven factors over a defined period of time with long-term outcomes across human, animal and ecosystem health. It does this while being cognizant of the need to assess and consider both co-benefits and trade-offs when designing and implementing systems, services, programmes and interventions that are multisectoral and One Health in nature. It incorporates all the major themes that have emerged from our evidence synthesis that are critical to a One Health conceptualization of sustainability. The key themes include ensuring 1) quality and evidence-based best-practice for any One Health initiatives, 2) social, economic and environmental viability across systems and sectors, 3) that processes are initiated and implemented equitably and justly while effective and are adapted as required (including where appropriate discontinued) over defined periods of time, 4) a state of optimized and balanced positive health and well-being outcomes are produced across humans, animals and ecosystems by accounting for co-benefits and trade-offs between sectors, 5) these processes and outcomes are considered over the long-term without having an adverse impact on the health and well-being of future generations. The definition for sustainability across the One Health paradigm reads as follows:

*A quality-assured system, strategy, service, programme, project, intervention or initiative, which is equitably and justly delivered while being socially, economically and environmentally viable, and that continues to be implemented while effective, adapting as needed over a defined period of time, to produce ****a state of*** *optimized health benefits across humans, animals and ecosystems without compromising the ability of future generations to meet their health and well-being needs.*

### Limitations

While our study followed best practice for systematic reviews in line with PRISMA guidelines, there were some limitations to our findings. Methodologically, the wide heterogeneity of definitions and terminology across sectors mean that some potentially useful studies may have been missed because sustainability focused review studies in those sectors (particularly animal health and environment sciences) are not always framed around health-related processes or outcomes. The conceptual and largely qualitative focus of many of the review studies identified, also meant there was wide heterogeneity in the quality of studies included as identified through our AMSTAR 2 appraisal (appendix 3). In keeping with our inclusive conceptual remit, we did not rank or weight determinants and characteristics of sustainability according to review quality; instead, we present them as a consolidated map of what has been proposed in the published review literature, noting that their relative importance is likely to be context-specific. These gaps highlight the methodological challenges of disentangling complex system interactions across sectors and disciplines that each have their own jargon and epistemic tradition.

We also recognise that restricting inclusion to English-language full texts may have led to the under-representation of conceptual and evaluative work produced in non-Anglophone high income settings as well as low- and middle-income country contexts, particularly in francophone and lusophone Africa and parts of Latin America and Asia. This is an important limitation for One Health, and we therefore interpret our findings as a synthesis of predominantly English-language review literature, rather than a comprehensive account of all global perspectives.

## Conclusion

Our umbrella systematic review brings together research and evidence reviews on sustainability and health from across the spectrum of One Health relevant disciplines. The integrated definition and meta-conceptual framework for analysis developed is intended to foster a shared understanding of sustainability across health-related disciplines and sectors, and encourage both communication and collaboration. Future research should include both quantitative and qualitative studies to evaluate and validate the framework and the identified characteristics of sustainability with all stakeholders in different One Health relevant contexts and settings. Empirical validation may include applying the framework to retrospective programme analyses, prospective implementation studies, participatory systems mapping with multisectoral stakeholders, or embedding the model within evaluation designs for One Health programmes to test construct validity and practical usability. Its potential utility at different scales, segments and levels of society also needs to be examined and tested across the varying set of health challenges at the human-animal-environment interface. There should also be increased focus on the process determinants and metrics for sustainability in the animal and environmental sectors especially to address some of the power imbalances and inequalities our review has found. Our integrated definition and framework offer a practical scaffold for policymakers to embed sustainability considerations explicitly into programme design, evaluation protocols and cross-sector governance instruments, particularly as countries develop and operationalize national One Health strategies and initiatives.

In an increasingly strained funding environment globally for health and the development sector more broadly, the need to ensure sustainability and consolidate capacity building efforts around the most effective, equitable and impactful interventions is critical. With the global geopolitical order also in constant upheaval and flux, with even well-established international organs like the WHO and the implementation of its related legal agreements for health now under threat, long held assumptions and constants around global health are now under threat of unravelling. This underscores the importance of appreciating that any measure or conceptualisation of sustainability is ultimately a function of time, subject to all its uncertainty, and that nothing remains immutable or unchanging.

## Electronic supplementary material

Below is the link to the electronic supplementary material.

Supplementary Material 1

Supplementary Material 2

Supplementary Material 3

## Data Availability

All data generated or analysed during this study are included in this published article [and its supplementary information files].

## Abbreviations

3P’s: Products, Pollutants, Pathogens
AMR: Antimicrobial Resistance
AMSTAR 2: A MeaSurement Tool to Assess Systematic Reviews
COVID-19: Corona Virus Disease of 2019
M&E: Monitoring and Evaluation
PRISMA: Preferred Reporting Items for Systematic reviews and Meta-Analyses
SDG: Sustainable Development Goals
UN: United Nations
